# The inhibition of thymidine phosphorylase can reverse acquired 5FU-resistance in gastric cancer cells

**DOI:** 10.1007/s10120-018-0881-3

**Published:** 2018-10-01

**Authors:** Ryutaro Mori, Kazuhiro Yoshida, Manabu Futamura, Tomonari Suetsugu, Kaoru Shizu, Toshiyuki Tanahashi, Yoshihiro Tanaka, Nobuhisha Matsuhashi, Kazuya Yamaguchi

**Affiliations:** Department of Surgical Oncology, Gifu University Graduate School of Medicine, 1-1 Yanagido, Gifu, 501-1194 Japan

**Keywords:** Stomach neoplasms, 5FU, Drug resistance, Thymidine phosphorylase

## Abstract

**Background:**

5FU can be converted to its active metabolite fluoro-deoxyuridine monophosphate (FdUMP) through two pathways: the orotate phosphoribosyl transferase–ribonucleotide reductase (OPRT–RR) pathway and the thymidine phosphorylase–thymidine kinase (TP–TK) pathway. We investigated the mechanism underlying 5FU-resistance, focusing on the changes in the 5FU metabolisms.

**Methods:**

MKN45 and 5FU-resistant MKN45/F2R cells were treated with 5FU or fluoro-deoxyuridine (FdU) in combination with hydroxyurea (HU) or tipiracil (TPI). The amount of FdUMP was determined by the density of the upper band of thymidylate synthase on Western blotting.

**Results:**

The MKN45/F2R cells exhibited 5FU resistance (37.1-fold) and showed decreased OPRT and increased TP levels. In both cells, the FdUMP after treatment with 5FU was decreased when RR was inhibited by HU but not when TP was inhibited by TPI. A metabolome analysis revealed the loss of intracellular deoxyribose 1-phosphate (dR1P) in both cells, indicating that FdUMP was synthesized from 5FU only through the OPRT–RR pathway because of the loss of dR1P. After the knockdown of TK, the FdUMP after treatment with FdU was decreased in MKN45 cells. However, it was not changed in MKN45/F2R cells. Furthermore, TP inhibition caused an increase in FdUMP after treatment with 5FU or FdU and reversed the 5FU resistance in MKN45/F2R cells, indicating that FdUMP was reduced through the TP–TK pathway in MKN45/F2R cells.

**Conclusions:**

In MKN45/F2R cells, the reduction of FdUMP through the TP–TK pathway caused 5FU resistance, and the inhibition of TP reversed the resistance to 5FU, suggesting that the combination of 5FU and TPI is a promising cancer therapy.

## Introduction

Gastric cancer remains a major cause of cancer death worldwide [1], as many patients with gastric cancer are still diagnosed only at late stages, and recurrent tumors are often detected even after curative surgery. Therefore, the development of drug therapies for gastric cancer is very important.

5FU is currently a key drug for both adjuvant therapy after curative operation [2–4] and for metastatic gastric cancer [5, 6]. Three mechanisms have been proposed for its action: incorporation into RNA [7], incorporation into DNA [8], and the inhibition of thymidine synthase (TS) leading to the inhibition of DNA de novo synthesis [9]. Although the incorporation into RNA and DNA is certainly important for understanding the mechanism of action of 5FU, the potential mechanism that has received the most focus is the inhibition of TS, as many chemotherapeutic drugs similarly inhibit TS, such as methotrexate, pemetrexate, and raltitrexed [10, 11].

**Fig. 1:**
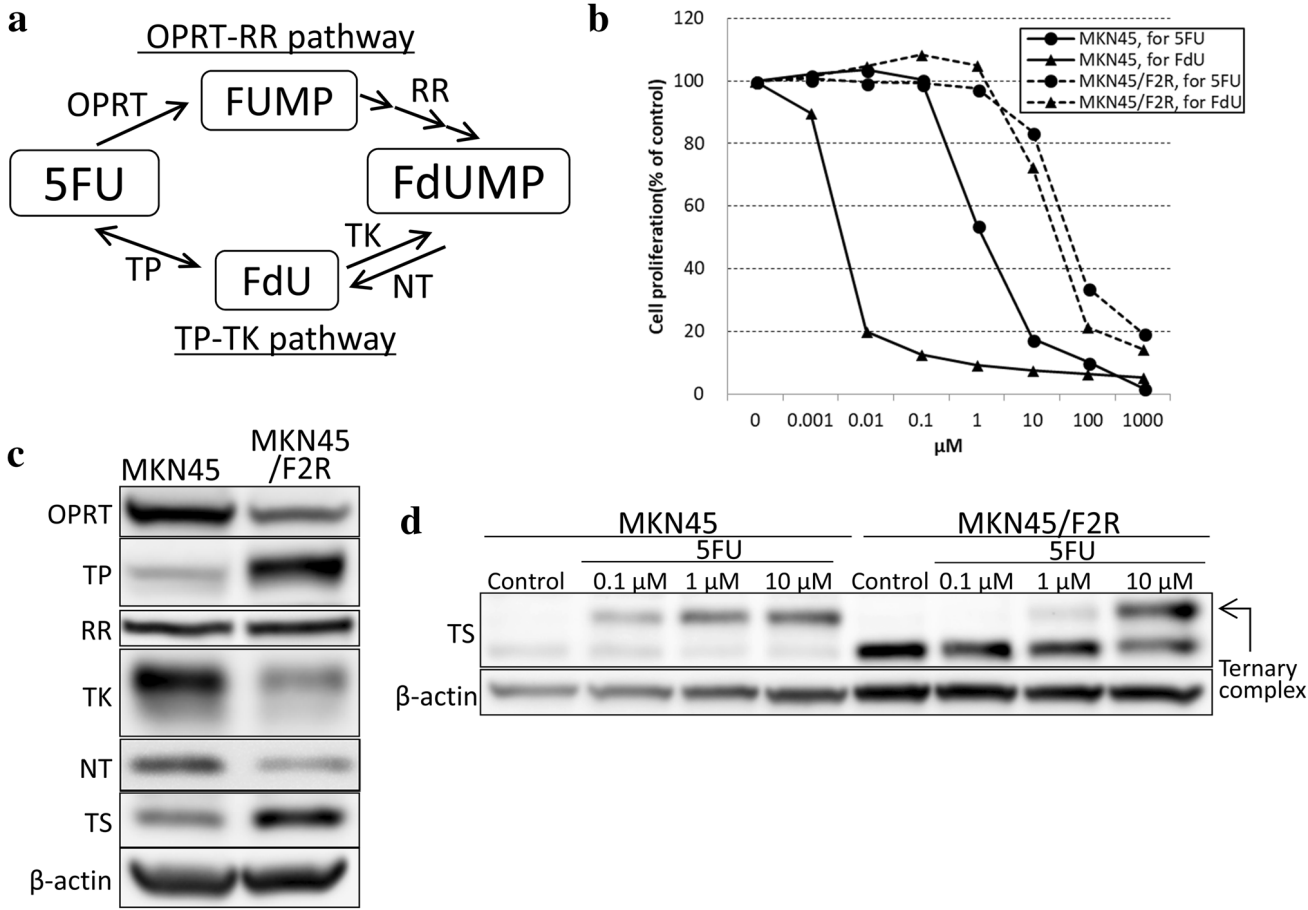
Metabolism for 5FU and sensitivities to 5FU and FdU in MKN45 and MKN45/F2R cells. **a** Diagram for 5FU metabolism. **b** An MTT assay for 5FU and FdU. **c** A Western blot analysis of the enzymes for 5FU metabolism. **d** A Western blot analysis of TS after treatment with 5FU. *FUMP* fluorouridine monophosphate, *FdU* fluoro-deoxyuridine, *FdUMP* fluoro-deoxyuridine monophosphate, *OPRT* orotate phosphoribosyl transferase, *TP* thymidine phosphorylase, *RR* ribonucleotide reductase, *TK* thymidine kinase, *NT* nucleotidase, *TS* thymidylate synthase

5FU is converted to fluoro-deoxyuridine monophosphate (FdUMP) in cancer cells and inhibits TS by forming a ternary complex composed of TS, 5,10-methylenetetrahydrofolate (CH2THF), and FdUMP [12]. Therefore, FdUMP is the key molecule in this mechanism. There are two speculated pathways for synthesizing FdUMP: (1) 5FU is converted to 5-fluorouridine monophosphate (FUMP) by orotate phosphoribosyltransferase (OPRT) and then converted to FdUMP by several enzymes, including ribonucleotide reductase (RR), in a process known as the “OPRT–RR pathway”; or (2) 5FU is converted to fluoro-deoxyuridine (FdU) by thymidine phosphorylase (TP) and then converted to FdUMP by thymidine kinase (TK), in a process known as the “TP–TK pathway”. These mechanisms are illustrated in Fig. 1a.

As mentioned above, decreased levels of OPRT, decreased TP and increased levels of TS can be considered as major factors involved in the development of 5FU resistance [13]. However, analyses of the gene expression in patients enrolled in the ACTS-GC trial, which investigated the efficacy of S-1 (5FU derivatives) for adjuvant therapy, revealed that increased TS was, if anything, related to a good prognosis, and that the expressions of OPRT and TP were not related to the prognosis at all [14].

These findings suggest that there may be unknown mechanisms of action involved in the development of 5FU resistance. Therefore, we investigated the mechanism of 5FU resistance using 5FU-resistant gastric cancer cell lines focusing on the changes of metabolisms for 5FU.

## Materials and methods

### Drugs

5FU was kindly provided by Kyowa Hakko (Tokyo, Japan). FdU, hydrochloride (TPI), and hydroxyurea (HU) were purchased from Sigma-Aldrich (St. Louis, MO, USA).

### Cell lines and cell culture

MKN45 cells (a poorly differentiated gastric cancer cell line, kindly provided by Hiroshima University, Hiroshima, Japan) were cultured in RPMI-1640 with 5% fetal bovine serum (FBS; both Wako Pure Chemical Industries, Ltd., Osaka, Japan) and sodium pyruvate (Sigma-Aldrich; Merck KGaA). MKN45/F2R cells are a 5FU-resistant cell line that was established by continuously exposing MKN45 cells to increasing concentrations (0.1–2 µM) of 5FU over a year, as previously described [15]. These cells were routinely maintained in RPMI-1640 with 5% FBS containing 2 µM 5FU, and prior to the study, the resistant cells were cultured in drug-free RPMI-1640 with 5% FBS for at least 2 weeks to eliminate the effects of 5FU in the experiments. The two cell lines were incubated in a humidified atmosphere of 5% CO_2_ at 37 °C.

### Western blot analyses and antibodies

The cells were lysed in RIPA buffer (Sigma-Aldrich; Merck KGaA) for 15 min on ice. The protein concentration of the lysates was measured using a Bio-Rad Protein Assay Dye Reagent Concentrate (Bio-Rad Laboratories, Inc., Hercules, CA, USA). The cell lysates were boiled in sample buffer solution (Wako Pure Chemical Industries, Ltd.). Total cell protein extracts (10 µg/lane) were separated by 10% SDS-PAGE using SuperSep™ ACE (Wako Pure Chemical Industries, Ltd.) and electrophoretically transferred onto polyvinyl difluoride (PVDF) membranes (EMD Millipore, Billerica, MA, USA). The membranes were blocked with PVDF blocking reagent (Toyobo Co., Ltd., Osaka, Japan) for 1 h. The membranes were then incubated with primary antibodies, such as β-actin (13E5) Rabbit mAb #4970 (Cell Signaling Technology, Danvers, MA, USA; 1:5000), RRM1 (D12F12) XP Rabbit mAb #8637 (Cell Signaling Technology; 1:5000), Anti-Thymidine Kinase 1 [EPR3193] antibody (ab76495) (Abcam, Cambridge, MA, USA; 1:50,000), Rabbit polyclonal to Thymidine Phosphorylase (ab69120) (Abcam; 0.4 µg/ml), dNT-1 (C-10): sc-390041 (Santa Cruz Biotechnology, Dallas, TX, USA; 1:100), Anti-Thymidylate Synthase, clone TS106 (MAB4130) (EMD Millipore, Billerica; 1:5000), or Anti-OPRT antibody (kindly provided by Taiho Pharmaceutical Company, Tokyo, Japan; 1:10,000) for 2 h at room temperature. The primary antibodies were diluted with Can Get Signal Solution 1 (Toyobo Co., Ltd., Osaka, Japan). The membranes were then washed with Dako Washing Buffer (Agilent Technologies, Inc., Santa Clara, CA, USA) and incubated with Goat anti-Mouse IgG, Peroxidase Conjugated, heavy chain + light chain (AP124P) (EMD Millipore) or Goat anti-Rabbit IgG, Peroxidase Conjugate (AP132P) (EMD Millipore) diluted to 1:25,000 with Can Get Signal Solution 2 (Toyobo Co., Ltd.) for 1 h at room temperature. Immunoreactive proteins were visualized with the ImmunoStar LD reagent (Wako Pure Chemical Industries, Ltd.), and images were captured using a GeneGnome HR system (Syngene Europe, Cambridge, UK).

### 3-(4,5-Dimethyl-2-tetrazolyl)-2,5-diphenyl-2H tetrazolium bromide (MTT) assay for the effects of 5FU, FdU or TPI

A total of 5 × 10^3^ cells were seeded into each well of 96-well plates and cultured for 24 h at 37 °C. The cells were treated with 5FU, FdU or TPI for 72 h, after which the culture medium was removed and 100 µl of a 0.5 mg/ml solution of MTT (Sigma-Aldrich; Merck KGaA) was added to each well. The plates were then incubated for 4 h at 37 °C. The MTT solution was replaced with 100 µl of dimethyl sulfoxide (Wako Pure Chemical Industries, Ltd.) per well, and the absorbance at 540 nm was measured using a Sunrise Rainbow RC-R (Tecan Group Ltd. Männedorf, Switzerland). Each assay was repeated eight times.

### Metabolomics analyses for phosphoribosyl pyrophosphate (PRPP) and deoxyribose 1-phosphate (dR1P)

Metabolomics analyses were conducted by Human Metabolome Technologies (Yamagata, Japan). Therefore, the samples were prepared according to their instructions. In brief, MKN45 cells and MKN45/F2R cells were disseminated to six 10 cm dishes and cultured for 24 h. Half of them were then treated with 2 µM of 5FU for 24 h. Culture media were removed and washed by PBS buffer and treated with supplied reagents. The supernatant was ultrafiltrated using the supplied filters, and frozen at − 80 °C. These samples were then transferred on dry ice.

### Transfection and small interfering RNA experiments for OPRT and TK1

MKN45 cells were cultured in RPMI-1640 with 5% FBS without antibiotics for 24 h to 50%–70% confluence prior to transfection. Cells were then transfected with siRNA oligonucleotides using Lipofectamine RNAiMAX (Invitrogen; Thermo Fisher Scientific, Inc., Waltham, MA, USA) and serum-free Opti-MEM (Invitrogen; Thermo Fisher Scientific, Inc.) at a final concentration of 10 nmol/l siRNA for 48 h. siRNA oligonucleotides for OPRT (Stealth RNAi; cat. no. 10620319-281527 A02 and 10620318-281527 A03), TK1 (Silencer Select RNAi; cat. no. s14160 and s14159) and negative control oligonucleotides (Stealth RNAi siRNA Negative Control; cat. no. 452002) were purchased from Invitrogen (Thermo Fisher Scientific, Inc.).

### Statistical analyses

The mean half maximal inhibitory concentration (IC50) values were calculated based on each result of the MTT assays using the Microsoft Excel 2010 software program (Microsoft Corporation, Redmond, WA, USA) and presented as mean ± standard error (SE). The significance of differences in the results of metabolomics analyses was determined by Human Metabolome Technologies using Student’s *t* test.

## Results

### Sensitivities to 5FU and FdU and the changes in the expression of the enzymes for 5FU metabolism in MKN45 and MKN45/F2R cells were assessed

MKN45/F2R cells that had been previously established as 5FU-resistant cells showed an IC50 of 50.4 ± 2.52 µM, which represented a 37.1-fold increased resistance compared with parental MKN45 (IC50: 1.36 ± 0.24 µM). MKN45/F2R cells also exhibited resistance to FdU (IC50: 28.8 ± 11.0 µM). Furthermore, parental MKN45 cells were very sensitive to FdU (IC50: 0.004 ± 0.000 µM). These results are shown in Fig. 1b. A Western blot analysis showed decreased OPRT (44.7%), TK (50.0%) and NT (43.4%) and increased TP (4.03-fold) and TS (1.83-fold) in MKN45/F2R cells compared with parental MKN45 cells (Fig. 1c). After 5FU treatment, both cell lines exhibited upper bands of TS on a Western blot analysis (Fig. 1d), which represents TS in ternary complexes composed of TS, CH2THF and FdUMP; the density of the upper band is correlated with the intracellular concentration of FdUMP [16]. MKN45/F2R cells exhibited a decreased upper band compared with parental MKN45 cells, which indicated that the formation of FdUMP from 5FU was decreased in MKN45/F2R cells. Based on these results, we used 0.1 µM of 5FU for MKN45 cells and 1 µM of 5FU for MKN45/F2R cells to make the density of the upper band of TS uniform for both cell lines.

### FdUMP was synthesized only through the OPRT–RR pathway due to the loss of intracellular dR1P, which is required for the TP–TK pathway

Hydroxyurea is the inhibitor for RR, and TPI is the inhibitor for TP. We investigated the changes in the amount of FdUMP after treatment with 5FU combined with HU or TPI to clarify which pathway is important for the synthesis of FdUMP.

**Fig. 2:**
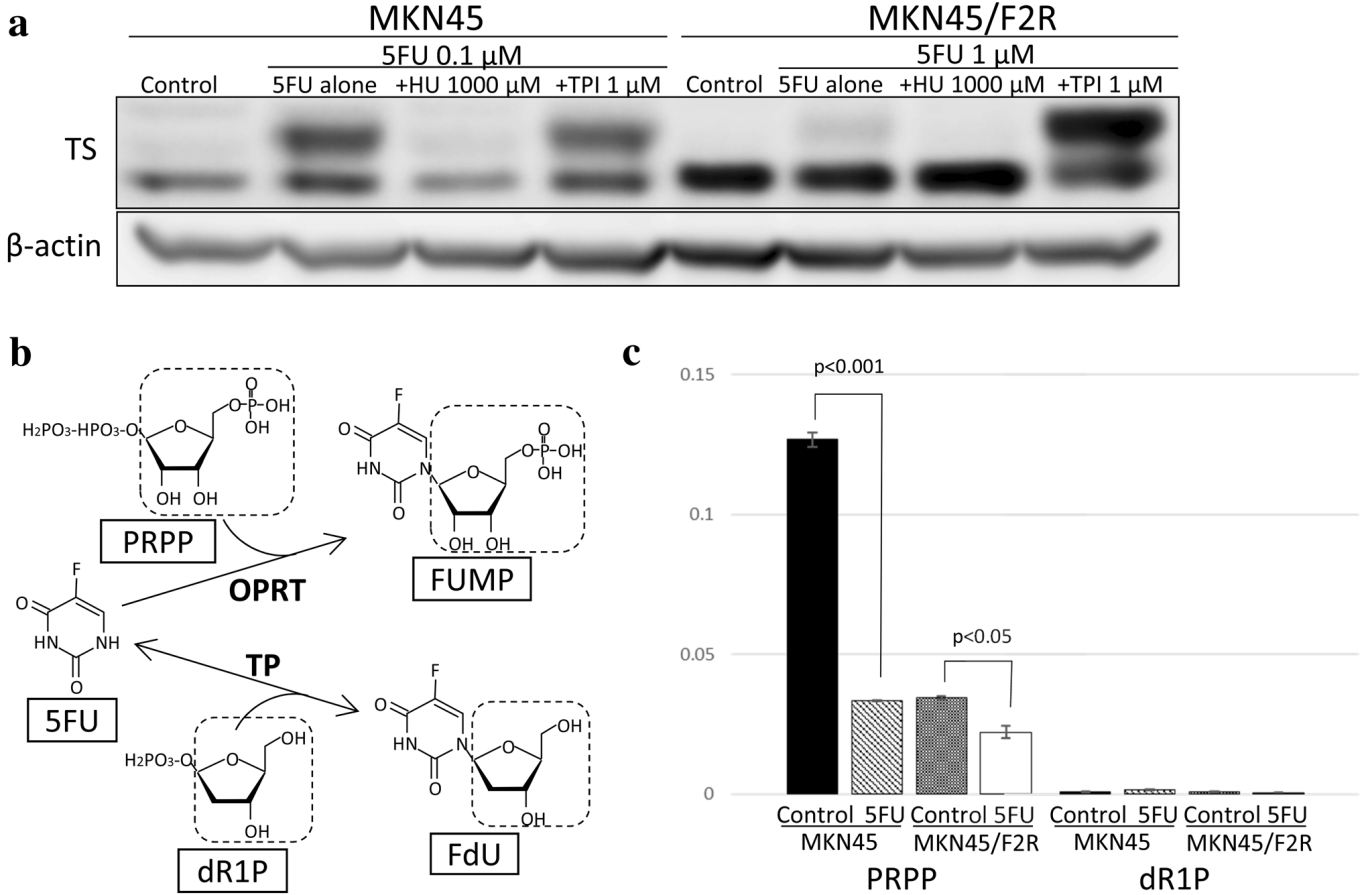
Changes in the amount of FdUMP after treatment with 5FU when RR or TP was inhibited. **a** A Western blot analysis of TS after treatment with 5FU with/without HU or TPI. **b** Diagram of 5FU metabolism by OPRT or TP. **c** The amounts of PRPP and dR1P after treatment with 5FU measured by a metabolome analysis. *HU* hydroxyurea, *TPI* thymidine phosphorylase inhibitor, *PRPP* phosphoribosyl pyrophosphate, *dR1P* deoxyribose 1-phosphate

As shown in Fig. 2a, both cell lines exhibited decreased upper band of TS (MKN45: 16.1%, MKN45/F2R: 46.3%) after treatment with 5FU combined with 1000 µM of HU, which indicated that FdUMP was synthesized through the OPRT–RR pathway. However, the upper band of TS was not decreased after treatment with 5FU with 1 µM of TPI in either cell line. Interestingly, the upper band of TS in the 5FU-resistant MKN45/F2R cells was markedly increased (7.47-fold) compared with that of 5FU alone. These results suggested that FdUMP was synthesized only through the OPRT–RR pathway.

Next, we focused on the amounts of metabolomes, such as phosphoribosyl pyrophosphate (PRPP) and deoxyribose 1-phosphate (dR1P), to clarify the cause of no progression in the TP–TK pathway. PRPP is the metabolome required for the synthesis of fluorouridine monophosphate (FUMP) from 5FU by OPRT, and dR1P is required for the synthesis of FdU from 5FU by TP. These pathways are illustrated in Fig. 2b. The amount of PRPP in MKN45/F2R cells as measured by the metabolomics analysis was lower than that of parental MKN45 cells, and PRPP was significantly decreased after treatment with 5FU in both cell lines. Meanwhile, the amount of dR1P was very low—almost at the detection limit—in both cell lines (Fig. 2c). These results suggested that FdUMP could not be synthesized through the TP–TK pathway because of the very low amount of dR1P.

### Knockdown of OPRT reduced FdUMP, leading to resistance to 5FU in parental MKN45 cells

Reduced OPRT has been said to be a predictive factor for resistance to 5FU [15, 17], and FdUMP is synthesized only through the OPRT–RR pathway, as described above. Therefore, we investigated the influence of the knockdown of OPRT on the resistance to 5FU.

**Fig. 3:**
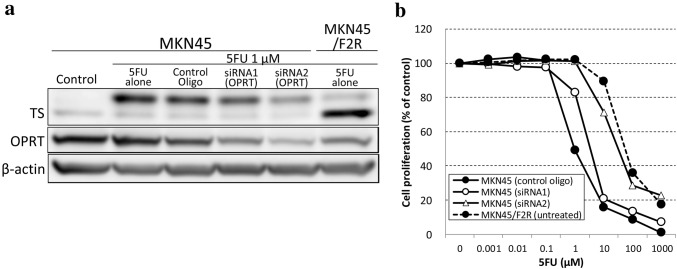
Decreased FdUMP and increased resistance to 5FU after knockdown of OPRT in MKN45 cells. **a** A Western blot analysis of TS and OPRT after treatment with 5FU when OPRT was knocked down. **b** An MTT assay for 5FU in MKN45 with the knockdown of OPRT and MKN45/F2R

As expected, the knockdown of OPRT by siRNA1 (OPRT) (59.6%) and siRNA2 (OPRT) (35.6%) caused a reduction in FdUMP after treatment with 5FU (76.8% and 48.1%, respectively) in parental MKN45 cells (Fig. 3a). Consistently, an MTT assay showed that the resistance to 5FU in MKN45 cells after transfection with siRNA1 (OPRT) and siRNA2 (OPRT) was increased (IC50: 3.53 ± 0.61 µM and 35.0 ± 3.10 µM, respectively) compared with MKN45 transfected with control oligo (IC50: 1.06 ± 0.168 µM) (Fig. 3b). These results suggested that reduced OPRT caused resistance to 5FU, although it was not the only cause of 5FU resistance.

### In MKN45/F2R, FdUMP after treatment with FdU was not synthesized by TK

Fluoro-deoxyuridine can be converted to FdUMP by TK because TK is the enzyme that converts nucleosides to nucleotides. Therefore, we investigated the changes in the metabolism of FdU after the knockdown of TK in both cells.

**Fig. 4:**
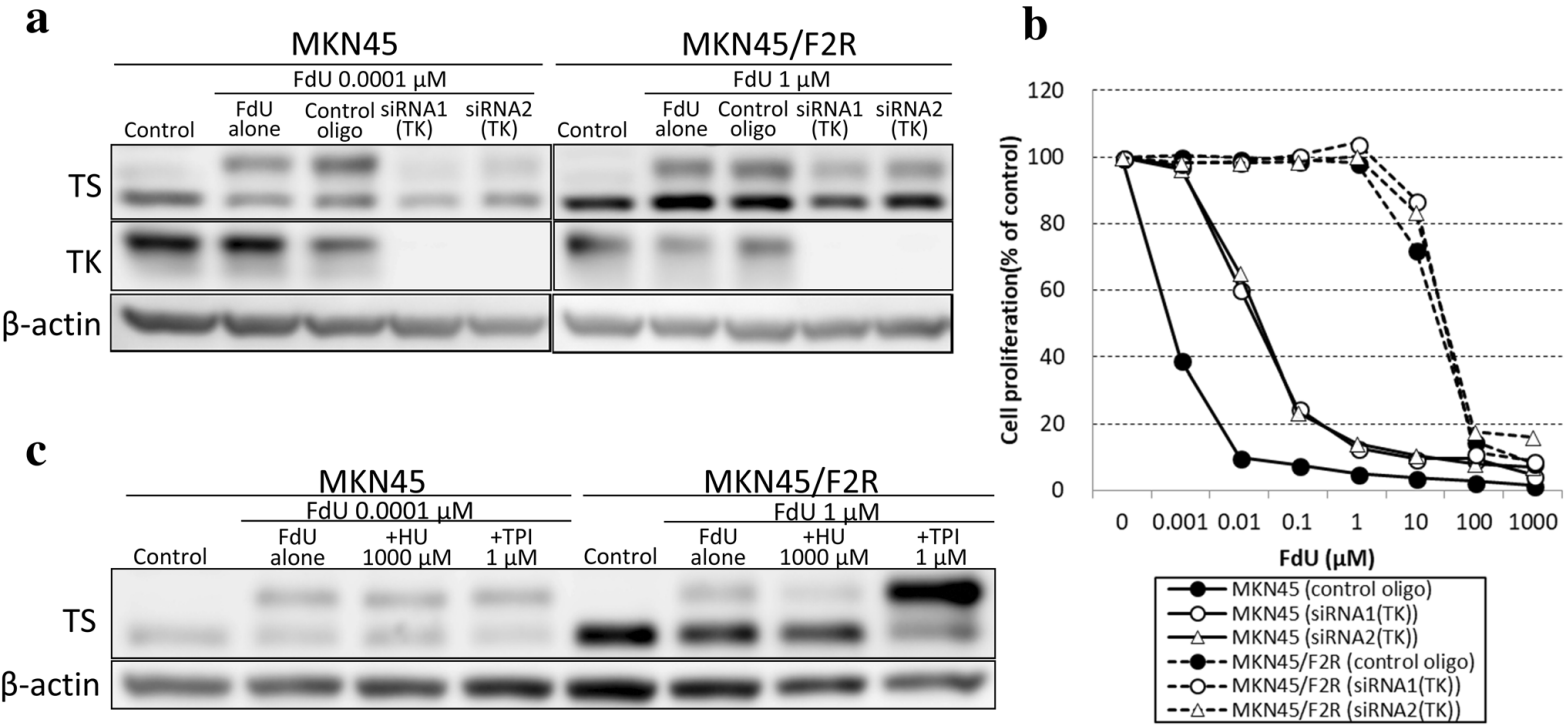
Changes in the amount of FdUMP after treatment with FdU and sensitivity to FdU when TK was knocked down. **a** A Western blot analysis of TS and TK after treatment with FdU when TK was knocked down. **b** An MTT assay for FdU in MKN45 and MKN45/F2R after the knockdown of TK. **c** A Western blot analysis of TS after treatment with 5FU with/without HU or TPI

When TK was knocked down by siRNA1 (TK) and siRNA2 (TK), the expression of TK almost disappeared on Western blotting, and the amount of FdUMP after treatment with FdU decreased (17.5% and 27.4%, respectively) in parental MKN45, indicating that FdU had been converted to FdUMP by TK in parental MKN45 cells. However, the amount of FdUMP was not decreased as much in 5FU-resistant MKN45/F2R cells after the knockdown of TK as in parental MKN45 cells (44.6% and 65.9%, respectively; Fig. 4a). Consistently, the resistance to FdU in parental MKN45 after the knockdown of TK by siRNA1 (TK) and siRNA2 (TK) was increased (IC50: 0.022 ± 0.003 µM and 0.024 ± 0.003 µM, respectively) compared with that in parental MKN45 cells transfected with control oligo (IC50 < 0.001 µM) and was almost unchanged in 5FU-resistant MKN45/F2R cells [IC50: siRNA1 (TK) 33.6 ± 3.11 µM, siRNA2 (TK) 33.9 ± 2.35 µM and control oligo 25.8 ± 2.86 µM, respectively; Fig. 4b]. We also investigated the changes in the amount of FdUMP after treatment with FdU by inhibiting RR or TP and found that the inhibition of RR by HU reduced the amount of FdUMP in MKN45/F2R cells (69.1%) but did not change the amount in MKN45 cells (92.6%) (Fig. 4c).

These results suggested that the catabolic reaction of TP for FdU was stronger than the anabolic reaction of TK for FdU in 5FU-resistant MKN45/F2R cells.

### 5FU resistance in MKN45/F2R was reversed by TP inhibition

As described above, the combination of TPI with 5FU increased the amount of FdUMP in 5FU-resistant MKN45/F2R cells. We therefore investigated the influence of TPI on the resistance to 5FU.

**Fig. 5:**
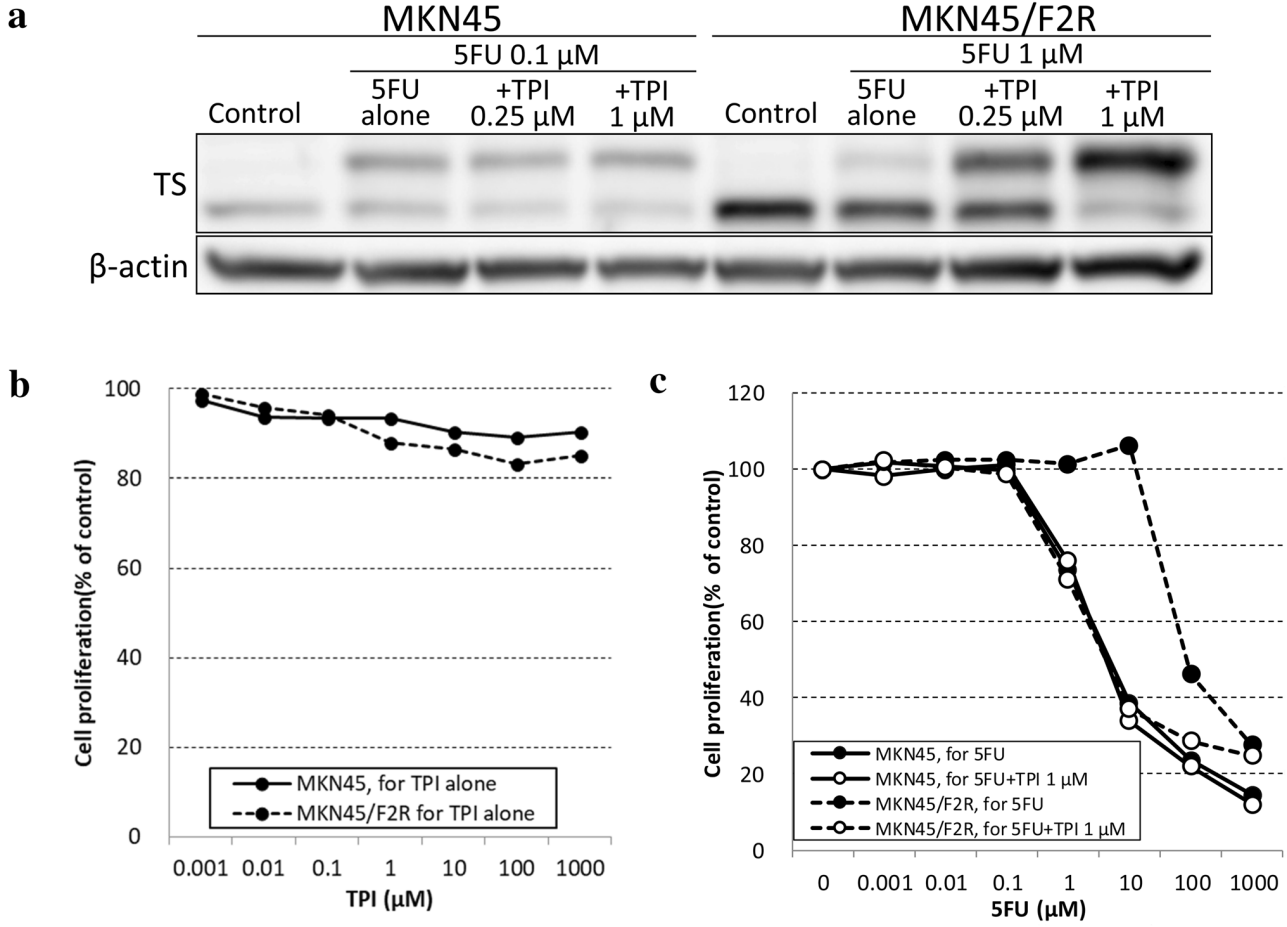
Reversal of 5FU resistance in MKN45/F2R by TPI. **a** A Western blot analysis of TS after treatment with 5FU and TPI. **b** An MTT assay for TPI alone. **c** An MTT assay for 5FU with/without 1 µM of TPI

In 5FU-resistant MKN45/F2R cells, the amount of FdUMP after treatment with 5FU was increased when 0.25 µM and 1 µM of TPI was combined (3.3- and 5.0-fold, respectively). However, parental MKN45 cells were not affected by the combination of TPI, as shown in Fig. 5a. On an MTT assay, TPI alone did not exert cytotoxicity in either cell line (Fig. 5b). Interestingly, the resistance to 5FU in 5FU-resistant MKN45/F2R cells was almost completely reversed when 1 µM of TPI was combined with 5FU (IC50: 72.28 ± 13.8 µM → 3.11 ± 0.36 µM), although the sensitivity to 5FU in parental MKN45 cells was not changed by TPI (IC50: 5.61 ± 6.86 µM → 3.47 ± 0.43 µM; Fig. 5c).

These results suggested that, in MKN45/F2R cells, intracellular FdUMP was reduced through the TP–TK pathway, which led to resistance to 5FU.

## Discussion

**Fig. 6:**
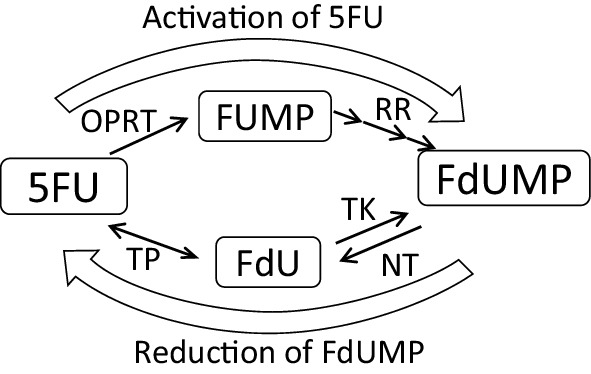
Diagram for the mechanism underlying the resistance to 5FU in 5FU-resistant cells. 5FU is activated only through the OPRT–RR pathway in both cell lines, and in 5FU-resistant cells, FdUMP is reduced through the TP–TK pathway

In the present study, we clarified that 5FU was converted to FdUMP only through the OPRT–RR pathway. Furthermore, intracellular FdUMP was reduced through the TP–TK pathway in 5FU-resistant MKN45/F2R cells, and the inhibition of TP led to the reversal of the resistance to 5FU in 5FU-resistant MKN45/F2R cells. These hypotheses are illustrated in Fig. 6.

In the present study, we mainly used common techniques, such as Western blot analyses, MTT assays and transfection of siRNA, and the only expensive technique used was a metabolomics analysis, which was conducted by an outside party. Therefore, it is easy for other researchers to repeat our study, and we believe that our results can be reproduced. However, to our knowledge, no study has investigated the mechanisms underlying 5FU resistance focusing on the changes in the metabolism of 5FU.

Thymidine phosphorylase has been believed to be an enzyme metabolizing 5FU in the first step, and several review articles have described the conversion of 5FU to FdU by TP [12]. TP is a phosphorylase, the reaction of which is usually reversible because of its chemical equilibrium [18]. Therefore, TP can theoretically convert 5FU to FdU. However, to our knowledge, no basic study has clearly described TP as an initial enzyme for 5FU under natural conditions, although some have described the conversion of 5FU to FdU by TP using TP-overexpressing cell lines [19]. Because dR1P cannot be synthesized de novo in the cell, 5FU does not appear to be converted to FdU by TP under natural conditions.

Orotate phosphoribosyl transferase has been reported as an important factor for 5FU resistance in cell lines, and there are many reports of the association between 5FU resistance and decreased OPRT levels [13, 15, 17]. In the present study, decreased OPRT also caused decreased intracellular FdUMP, leading to 5FU resistance. However, the resistance shown by MKN45/F2R cells could not be completely reproduced by the knockdown of OPRT alone. Of note, the combination of 5FU with TPI almost completely reversed the 5FU resistance, indicating that the reduction of FdUMP by the TP–TK pathway was a more influential factor for 5FU resistance than a decrease in OPRT.

We previously reported the reversal of 5FU resistance by the combination of 5FU with deoxyuridine (dU) [20]. In the present study, the intracellular concentration of FdUMP after treatment with 5FU in 5FU-resistant MKN45/F2R cells was also increased when a large amount (1000 µM) of dU was combined with 5FU. The enzymes associated with nucleotide metabolism often have a feedback mechanism, and excessive amounts of products generally inhibit the related enzymes. Therefore, dU seemed to inhibit nucleotidase (NT), which is one of the components of the TP–TK pathway. Because 1000 µM of dU cannot be practically applied, developing an NT inhibitor is expected to be another way of overcoming 5FU resistance.

TPI used in the present study has been already applied as a component of TAS-102, an anticancer drug used to treat metastatic colorectal cancer [21]. Therefore, the combination therapy with 5FU and TPI would be easy to incorporate, and we believe it will be a promising therapy for gastric cancer.

Several limitations associated with the present study warrant mention. In this study, the reversal of 5FU resistance by TPI was found only in MKN45/F2R cells. In addition, predictive markers for the reversal of 5FU resistance by TPI must be developed to apply combination therapy with 5FU and TPI in daily practice.

In conclusion, we observed the reversal of 5FU resistance in 5FU-resistant cells by TPI. Further investigations regarding the reversal of 5FU resistance by TPI in other 5FU-resistant cell lines as well as in vivo studies and the application of this combination therapy in clinical practice will be required in the future.
